# A Genomic Sequence Resource of *Diaporthe mahothocarpus* GZU-Y2 Causing Leaf Spot Blight in *Camellia oleifera*

**DOI:** 10.3390/jof10090630

**Published:** 2024-09-03

**Authors:** Xulong Shi, Yu Zhang, Jing Yang, Yunze Chen

**Affiliations:** 1 College of Forestry, Guizhou University, Huaxi District, Guiyang 550025, China; gs.xlshi23@gzu.edu.cn (X.S.); gs.yzhang22@gzu.edu.cn (Y.Z.); 2 School of Biological Sciences, Guizhou Education University, Wudang District, Guiyang 550018, China

**Keywords:** *Diaporthe mahothocarpus*, fungal genetics, gene annotation, pathogenicity

## Abstract

*Diaporthe mahothocarpus* GZU-Y2, a new pathogen responsible for leaf spot blight disease, leads to significant damage and economic losses in some *Camellia oleifera* plantations. The current study annotated the genome of the *D. mahothocarpus* isolate GZU-Y2 to advance our knowledge of the pathogen and facilitate improved disease management of leaf spot blight. The initial Pacbio-Illumina hybrid draft genome for GZU-Y2 resulted in a high-quality assembly with 62 contigs, characterized by an N50 length of 7.07 Mb. The complete genome of isolate GZU-Y2 was 58.97 Mbp, with a GC content of 50.65%. Importantly, the assembly exhibits remarkable integrity, with 97.93% of complete BUSCO validating genome completeness. The prediction results showed that a total of 15,918 protein-coding genes were annotated using multiple bioinformatics databases. The genome assembly and annotation resource reported here will be useful for the further study of fungal infection mechanisms and pathogen–host interaction.

## 1. Introduction

*Camellia oleifera* C.Abel., a woody tree species that produces edible oil, is unique to China and is one of the four major woody oil tree species in the world. *C. oleifera* has an extremely wide distribution range in China, from the coastal hills in the southeast to the Yunnan-Guizhou Plateau in the west. In recent years, the *C. oleifera* industry in Guizhou Province has developed significantly, but due to the relatively weak research on pest control, the harm of pests and diseases on *C. oleifera* has become increasingly serious. Among them, leaf spot blight of *C. oleifera* caused by *Diaporthe* spp. is one of the most important diseases, which results in significant leaf and fruit loss, ultimately affecting the yield and quality of the oil [1,2]. The disease initially manifests as light yellow, nearly round, or irregular spots on the leaf margins. These spots later turn dark brown, with the appearance of light brown and many dark black spots. The boundary between the diseased and healthy parts is distinct. As the disease progresses, the leaf blade eventually dies and turns gray. *Diaporthe* spp. associated with leaf blight of *C. oleifera* were identified and characterized as *D. camelliae oleiferae*, *D. hunanensis*, *D. hubeiensis*, and *D. sojae* [3,4]. We previously identified the pathogen isolate in Guizhou Province as *D. mahothocarpus* GZU-Y2, belonging to the *D. eres* species complex [5].

The genus *Diaporthe* is a significant group of plant pathogenic fungi that belongs to the Diaporthaceae family, with a broad host range and geographical distribution [6]. The MycoBank database currently records 1346 *Diaporthe* species and over 1057 species of its asexual form, *Phomopsis*, which are found worldwide, particularly in tropical and subtropical ecosystems. They are commonly found in plants as phytopathogenic, endophytic, or saprophytic fungi [7]. For example, *D. ampelina* is a significant pathogen responsible for grapevine twig blight and leaf spot, which infects shoots and leaves of grapes, causing up to 30% yield loss in temperate regions [8]. *D. citri* could cause citrus rot in all major citrus production areas except in Europe [9,10]. Furthermore, *D. helianthi* was initially reported in the former Yugoslavia, which primarily causes sunflower stem ulcer disease [11,12]. In addition, due to its biological and chemical diversity, *Diaporthe* spp. is also a rich source of active natural products. It has been found that they can produce a variety of metabolites with novel structures, including polyketides, alkaloids, terpenoids, and anthraquinones, which exhibit significant anti-tumor, antimicrobial, and antioxidant activities [13].

High-throughput whole genome sequencing is an effective method for gaining a comprehensive understanding of strain-related properties at the gene level [14]. Thus, the present study involved sequencing, predicting assembly, and annotating high-quality genome sequences of *D. mahothocarpus*, aiming to provide a systematic analysis of its pathogenicity and interaction mechanisms with the host at the molecular level.

## 2. Materials and Methods

### 2.1. Fungal Material and Culture Conditions

*Diaporthe mahothocarpus* GZU-Y2 was isolated from leaf-spot-blight diseased leaves of *Camellia oleifera*, preserved at 4 °C on potato dextrose agar (PDA) medium in the Forest Pathology Laboratory of the College of Forestry, Guizhou University (Guiyang, China). It was cultured on PDA at 28 °C for 7 days before DNA extraction.

### 2.2. DNA Extraction

The mycelium of D. mahothocarpus GZU-Y2 was obtained from cultures grown in 100 mL of fresh potato dextrose broth (PDB) at 28 °C for 2 days. Mycelium was filtered through sterile filter paper and ground to a powder in liquid nitrogen. Genomic DNA was then extracted by SDS-based DNA extraction using Omega Fungal DNA Kit D3390-02 (Omega Bio-Tek, Inc., Norcross, GA, USA). The extracted DNA was separated by 1% agarose gel electrophoresis, stained with ethidium bromide (0.1 mg/mL), detected by UV transmission, and quantified by Qubit.

### 2.3. Genome Sequencing and Assembly

The total DNA was sequenced using the PacBio Sequel II single-molecule real-time (SMRT) sequencing platform from Beijing PacMark Biotechnology Co., Ltd. (Beijing, China). The low-quality reads were filtered, and the remaining reads were assembled into a gap-free isoform using SMRT Link v5.0.1. Segments were spliced based on the overlapping regions between reads, first splicing them into longer contiguous sequences (contigs), and then contigs were spliced into longer scaffolds that were allowed to contain gap sequences (gaps) by eliminating errors and gaps in the scaffolds and localizing these scaffolds to chromosomes. The circular consensus sequencing (CCS) reads were assembled using Hifiasm 0.12-r304 (Use the parameter -t 16 followed by some of the default parameters: -k 51 -w 51 -f 37 -D 5.0 -N 100 -r 3 -a 4 -m 10,000,000 -p 100,000 -n 3) software. Then the assembled genes were further corrected by Pilon 1.22 (Uses parameters -mindepth 0.1 -changes -fix bases) software using the transcriptome sequencing data to obtain a final genome with higher accuracy [15,16].

### 2.4. Phylogenetic Analysis

*D. mahothocarpus* GZU-Y2 was allowed to grow using bwa with selected other strains (*D. amygdali* CAA958, *D. caulivora* D57, *D. aspalathi* MS-SSC91, *D. destruens* F3, *D. batatatis* CRI 302-4, *D. longicolla* TWH P74, *Phomopsis vexans* PV4, *D. ampelina* DA912, *D. velutina* CJ32, *D. citri* Q7, *D. citri* NFHF-8-4, *D. citri* ZJUD14, *D. capsici* GY-Z16, *D. vaccinii* CBS 11857, *D. eres* CBS 160.32) were compared to the genome. Notably, the annotations of these used fungal genomes are publicly available. The three conditions are equal to 0 to filter out the pure heterozygotes, and then we used the vt normalized pair and normalized to REF/ALT. Finally, we used PhyML (version 20120412) (parameter: -m GTR -f m -v e -a e -o tlr -b 100) to build the tree and used the itol online tool for the drawing of the pictures.

### 2.5. Genome Prediction

LTR_FINDER v1.05 [17], MITE-Hunter (http://target.iplantcollaborative.org/mite_hunter.html, accessed on 17 April 2024) [18], RepeatScout v1.0.5 [19], and PILER-DF v2.4 [20] were used to construct a repeat sequence database. This database was merged with the Repbase database to create the final database [21]. RepeatMasker v4.0.6 software was then used to predict the repeat sequences [22]. Gene structure prediction was mainly achieved through ab initio prediction, homologous protein prediction, and transcriptome data prediction. The three prediction results were then integrated. Genscan (http://hollywood.mit.edu/GENSCAN.html, accessed on 17 April 2024) [23], Augustus v2.4 [24], GlimmerHMM v3.0.4 [25], GeneID v1.4 [26], and SNAP version 2006-07-28 [27] were used to make de novo predictions. GeMoMa v1.3.1 [28] was used for homologous protein prediction. Hisat2 v2.0.4 [29] and Stringtie v1.2.3 [30] were used to perform assembly based on reference transcripts [31] and TransDecoder v2.0 for Unigene sequence prediction. Finally, EVM v1.1.1 was used to integrate the prediction results, and PASA v2.0.2 was used to modify [32]. Transfer RNA (tRNA) and ribosome RNA (rRNA) genes were predicted using tRNAscan-SE v1.3.1 [33] and Infernal v1.1.1 [34], respectively. The whole genomes were scanned using GenBlastA v1.0.1 (use parameters -P blast -pg tblastn -p T -e 1e-5 -g T -f F -a 0.5 -r 10 -c 0.5 -s 0) after masking predicted functional genes. Putative candidates were then analyzed for non-mature and frame-shift mutations using GeneWise v2.2.0 (Use the parameter -both -pseudo). The secondary metabolism gene cluster was predicted using antiSMASH v6.0.0 [35,36].

### 2.6. Gene Function Annotation

The proteins were predicted and then compared using blast (e-value: 1 × 10^5^) against Nr, Swiss-Prot (SWISS-PROT protein knowledgebase, http://www.expasy.org/sprot/, accessed on 17 April 2024) [37], TrEMBL (http://www.ebi.ac.uk/embl/index.html, accessed on 17 April 2024), KEGG (https://www.genome.jp/kegg/, accessed on 17 April 2024) [38], and KOG (https://ftp.ncbi.nlm.nih.gov/pub/COG/KOG/, accessed on 17 April 2024) [39]. GO annotation was performed using Blast2go 2.5 (-annot with some of the default parameters in the property file: Annotation.goweight = 5, Blast.hitDescPosition = 5), while Pfam annotation was performed using hmmer 3.0 (-E 0.00001 -domE 0.00001 -noali -acc -notextw) [40]. Additionally, pathogenicity can be investigated by blasting [41,42] against CAZy, TCDB, PHI, CYPED, and DFVF databases and to predict the abundance of BGC. For subcellular localization, the subcellular localization information is one of the key features of protein function research; secretory proteins were detected by SignalP 4.0 (-f long -g png followed by some of the default parameters -s best -c 70), and after transmembrane proteins were filtered by TMHMM 2.0c (Direct access to protein sequences), the candidate secretory proteins can be obtained [43,44,45]. EffectorP 2.0 (-i pep.fa -o Effector.result -E Effector.pep) was used to further analyze the secreted protein to predict the effector protein [46,47,48].

### 2.7. Data Availability

This Whole Genome Shotgun project has been deposited at DDBJ/ENA/GenBank under the accession JBBYIC000000000 (BioProject PRJNA1098494; BioSample SAMN40910135). The version described in this paper is version JBBYIC 010000000, which includes the sequencing data and the annotation file.

## 3. Results

### 3.1. Genome Assembly and Genomic Characteristics

The whole genome sequencing of *D. mahothocarpus* GZU-Y2 generated more than 6.92 G CCS reads with 117.34×-fold genome coverage. After removing duplicates and low-quality reads, the predicted genome size is 58.97 Mbp with 50.65% GC content (Table 1). The overall assembly genomic characteristics for GZU-Y2 are summed up in Table 1. In brief, the genome of GZU-Y2 consisted of 62 contigs in total, with a contig length of 58,973,678 bp, an N50 length of 7,066,871 bp, and no gaps. The completeness of the genome assembly of GZU-Y2 was 97.93%, assessed using BUSCO (Benchmarking Universal Single-Copy Homologous Genes) v2.0 software with a single-copy orthologous gene library. Moreover, 97.24%, 0.69%, 0, and 2.07% of the BUSCOs were single-copy, duplicated, fragmented, and missed, respectively.

Moreover, the prediction of repetitive sequences for *D. mahothocarpus* GZU-Y2 resulted in 1,901,760 bp, with a repetitive sequence ratio of 3.22%. Notably, this value is likely an underestimate, as we have filtered out alleles shorter than 1 kb, which may contain additional repetitive sequences. The statistical results of the proportions of different types of repetitive sequence elements are shown in Table 2. The analysis revealed that 246 Class I retrotransposons and 153 Class II DNA transposons were among the disrupted repeat sequences (DRs). Among them, Class I retrotransposons comprised 20 LINEs, 98 LTRs, 57 PLEs, 11 TRIMs, and 60 Unknown elements, while Class II DNA retrotransposons included 5 Helitrons, 45 MITEs, 85 TIRs, 18 Unknown elements, 58 Potential Host Genes, and 738 SSRs.

### 3.2. Phylogenetic Analysis

**Figure 1:**
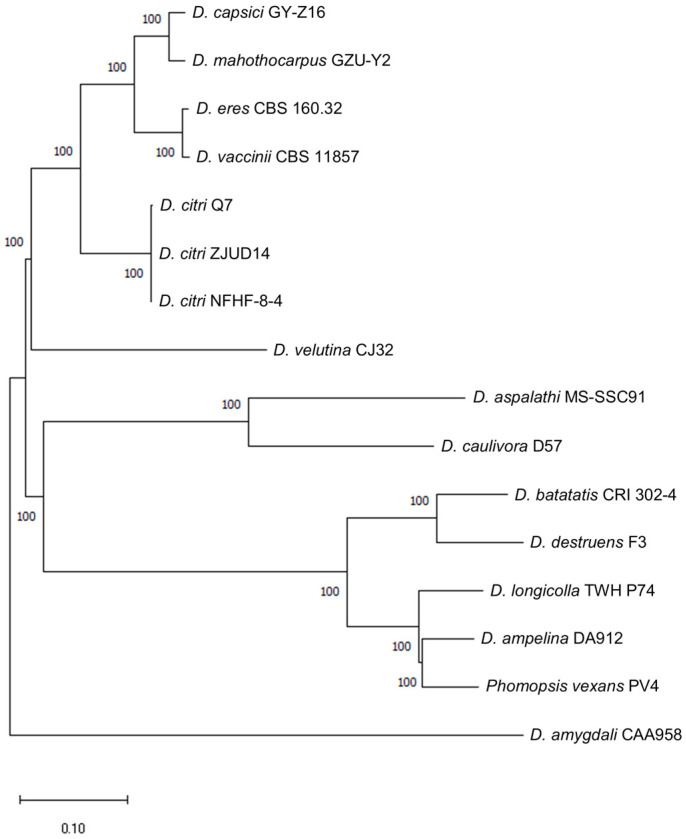
Phylogenetic tree constructed based on SNPs. Bar = 0.10.

By using snippy in snippy 4.6.0 for *D. amygdali* CAA958, *D. caulivora* D57, *D. aspalathi* MS-SSC91, *D. destruens* F3, *D. batatatis* CRI 302-4, *D. longicolla* TWH P74, *Phomopsis vexans* PV4, *D. ampelina* DA912, *D. velutina* CJ32, *D. citri* Q7, *D. citri* NFHF-8-4, *D. citri* ZJUD14, *D. capsici* GY-Z16, *D. vaccinii* CBS 11857, *D. eres* CBS 160.32 and *D. mahothocarpus* GZU-Y2 were analyzed separately, snippy-core analyses of the other strains and *D. mahothocarpus* GZU-Y2 as a whole were performed to obtain the results of each strain and the results of the overall analyses are shown in Figure 1.

### 3.3. Gene Prediction

#### 3.3.1. Prediction of Protein-Coding Genes

**Figure 2:**
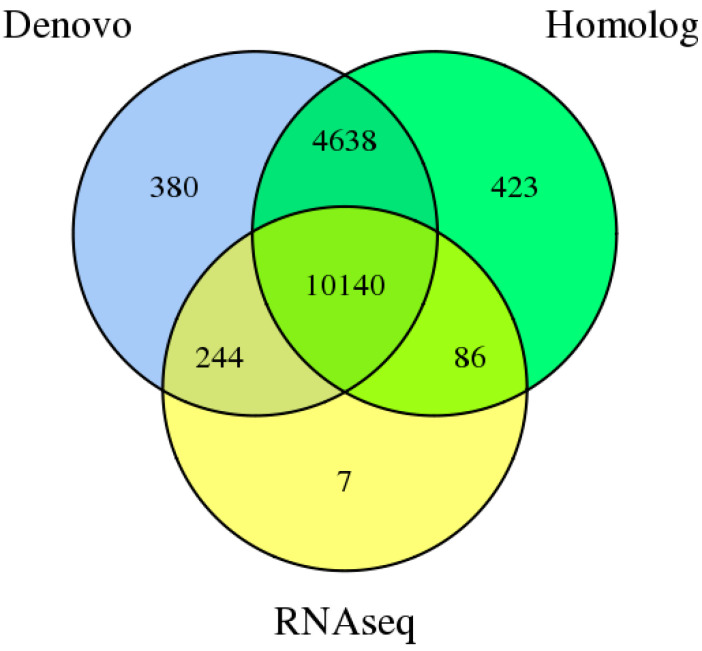
Integrated genes of *D. mahothocarpus* GZU-Y2 derived from three prediction methods.

The gene structure prediction of the *D. mahothocarpus* GZU-Y2 was based on de novo, homology, and transcriptome evidence. The results of the three types of predictions were integrated using EVM v1.1.1. Then, PASA v2.0.2 was used to modify the final 15,918 predicted genes, among which the number of genes supported by homology prediction and transcriptome prediction accounted for 97.61%, indicating a high quality of prediction (Figure 2). As seen from Table 3, the total length of the predicted genes was 33,450,242 bp, with an average length of 2101.41 bp. The number of CDS, introns, and exons are 46,210, 31,029, and 46,947, respectively.

#### 3.3.2. Prediction of Non-Coding RNA

Non-coding RNAs, which do not encode proteins, encompass a variety of RNAs with known functions, such as microRNAs, rRNAs, and tRNAs. Different strategies were employed to predict non-coding RNAs based on their structural characteristics, resulting in the prediction of 127 rRNAs, 361 tRNAs, and 89 other ncRNAs (Table 1).

#### 3.3.3. Prediction of Pseudogenes

The protein sequences predicted and obtained from the Swiss-Prot database were used to identify homologous gene sequences on the genome using GenBlastA software. Subsequently, the GeneWise software was used to identify premature termination codons and code-shifting mutations in the gene sequences, resulting in the identification of pseudogenes. A total number of 4 pseudogenes were predicted. The total length of the predicted pseudogene sequences was 282 bp, with an average length of 70.5 bp.

#### 3.3.4. Prediction of Gene Cluster

**Figure 3:**
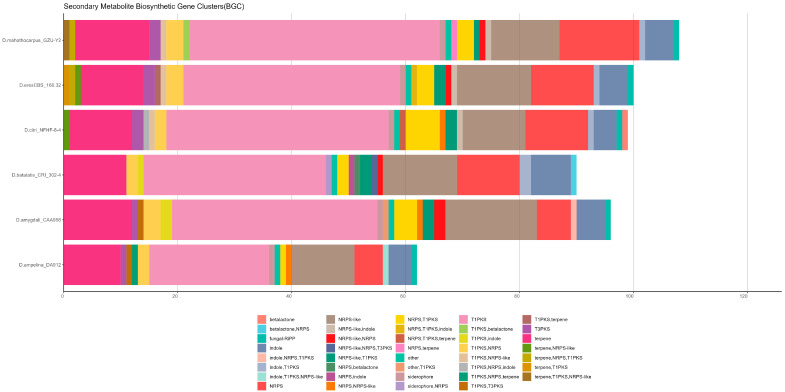
Cluster of biosynthetic genes identified in the analyzed Diaporthe genome.

The detection of *D. mahothocarpus* GZU-Y2 gene clusters was performed using an-tiSMASH v6.0.0, which predicted a total of 108 gene clusters with a combined length of 4,403,413 bp and an average length of 40,772 bp. Among them, there are 46 Type I polyketide synthases (T1PKSs) genes, 26 Non-ribosomal peptide synthetase (NRPs) genes, 5 indole genes, and 13 terpene genes. The specific information of gene clusters with alignment similarity greater than 80% is shown in Table 4. Among the genomes analyzed, *D. mahothocarpus* GZU-Y2 contained the highest number of BGCs (n = 108), followed by *D. eres* CBS 160.32 (n = 100), and the least was *D. ampelina* DA912 (n = 62). All the genomes analyzed were rich in T1PKS (terpene), NRPS (terpene), and terpene. Importantly, in *D. mahothocarpus* GZU-Y2, a gene cluster T1PKS (betalactone) was found, which was different and unique from the other genomes, and the results are shown in Figure 3. With its chemical diversity, betalactone is a good class of natural products with potential biological activities.

### 3.4. Gene Annotation

In the present study, 14,564 genes were annotated in the NCBI Nr database.

**Figure 4:**
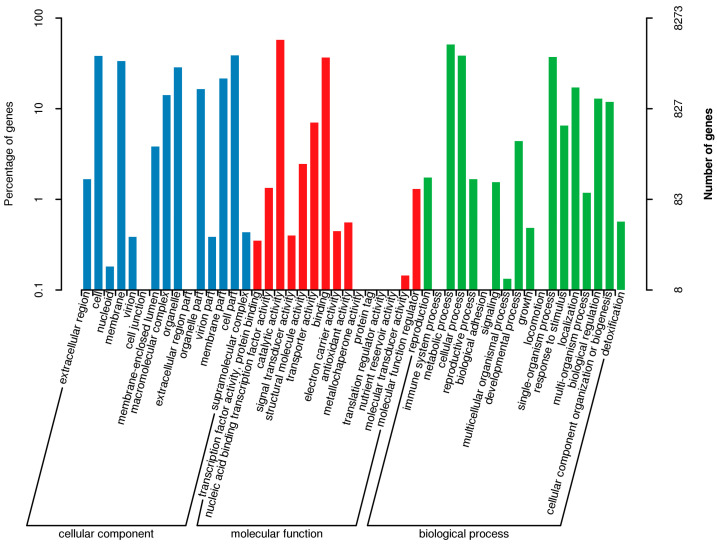
GO annotation of *D. mahothocarpus* GZU-Y2. The GO classification is presented with horizontal coordinates, while the percentage (**left**) and numbers (**right**) of genes are shown on the vertical axis.

The GO database categorizes gene functions into cellular components, molecular functions, and biological processes. A gene can be annotated multiple times through the GO program. The results of the GO database annotation of the *D. mahothocarpus* GZU-Y2 genome for the enrichment of genes for each secondary function of GO in the context of the total genes, reflecting the status of each secondary function in this context (Figure 4).

**Figure 5:**
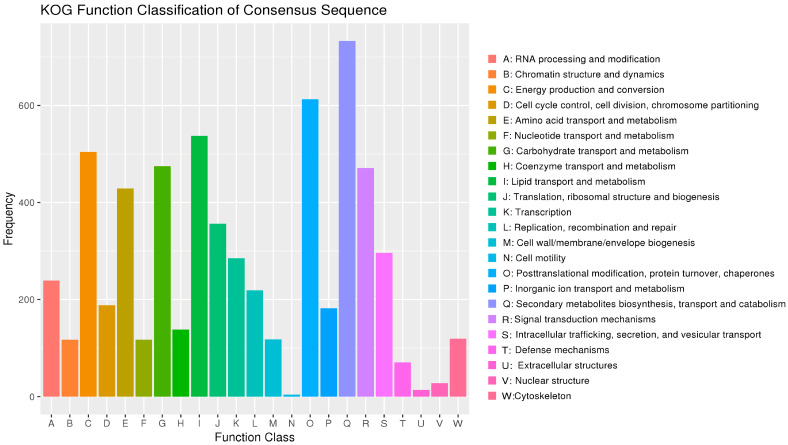
KOG annotation of *D. mahothocarpus* GZU-Y2. The horizontal axis represents the function class, and the vertical axis is the number of genes.

The KOG database is a collection of immediate homologous protein clusters from eukaryotic organisms. It is used to infer sequence functions through comparison and classification. The number of genes in different functional classes reflects the metabolic or physiological bias in the corresponding period and environment. This can be scientifically interpreted to determine the distribution of research subjects in each functional class (Figure 5).

**Figure 6:**
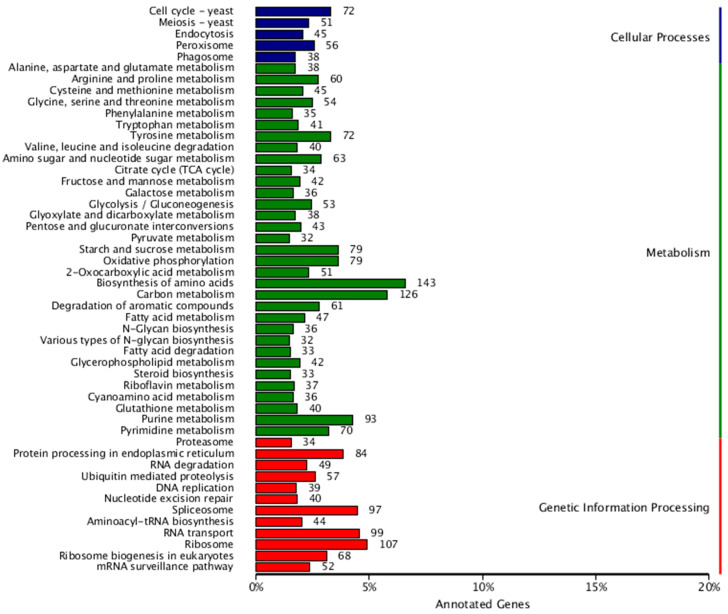
KEGG classification of *D. mahothocarpus* GZU-Y2. The left vertical axis is the KEGG level 3 classification, and the right is the KEGG level 1 classification. The horizontal axis is the percentage of annotated genes, and the label is the number of genes.

The KEGG database is a large knowledge base for systematically analyzing gene functions and linking genomic and functional information. The results showed that a total of 3750 genes were annotated, which were categorized into 3 major classes and 50 subclasses (Figure 6).

### 3.5. Carbohydrate-Active Enzymes (CAZymes)

**Figure 7:**
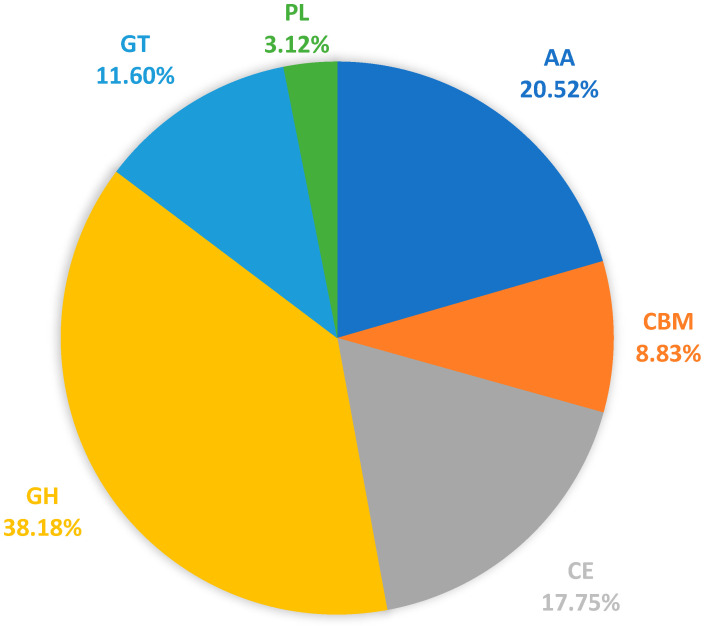
Predicted carbohydrate-active enzymes of *D. mahothocarpus* GZU-Y2. AA, auxiliary activities; GH, glycoside hydrolases; GT, glycosyl transferases; PL, polysaccharide lyases; CE, carbohydrate esterases; CBM, carbohydrate-binding modules.

**Figure 8:**
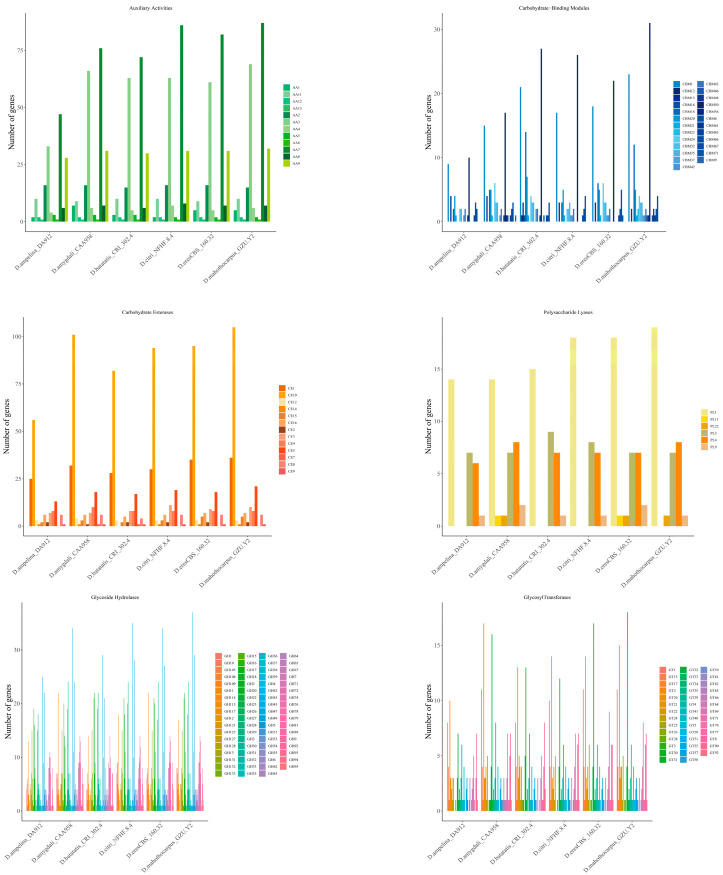
Number of predicted genes encoding for the most abundant carbohydrate-active enzyme families in all genomes of the analyzed *Diaporthe* species.

The CAZymes have a crucial role in breaking down complex carbohydrates and plant pathogenic fungi. Certain species of CAZymes are responsible for obtaining nutrients from plants and play a role in the infection and colonization process [49,50]. In *D. mahothocarpus* GZU-Y2, a total of 1155 CAZyme genes were identified, including 441 GHs, 134 GTs, 36 PLs, 205 CEs, 237 AAs and 102 CBMs (Figure 7). A total of 682 genes were identified to be involved in plant cell wall hydrolases, including GHs, PLs, and CEs. These enzymes are crucial for the successful penetration and infection of plant hosts by fungi. Overall, the number of CAZymes varied among species, with *D. mahothocarpus* GZU-Y2 and *D. eres* CBS 160.32 being the most abundant. Of all the categories of CAZymes detected, GHs and AAs were the two most predicted proteins, with AA3, AA7, AA9, CBM1, CBM50, CE10, CE1, GH109, GH16, GH18, GH3, GH43, GH5, GT2, GT32, and PL1 having an abundance in the different species analyzed. Among them, *D. mahothocarpus* GZU-Y2 had the highest content of CAZymes, including AA3, AA7, AA9, CBM1, CBM50, CE10, GH43 and PL1 families. It was followed by *D. eres* CBS 160.32, including AA3, AA7, CBM1, CBM50, CE10, GH16, GH43, GT32, and PL1 family, and the results are shown in Figure 8.

### 3.6. Pathogenic System Analysis

The Transporter Classification (TC) System, developed by TCDB, a database that classifies membrane transporter proteins, predicts a total of 135 genes related to transporter proteins in *D. mahothocarpus* GZU-Y2. The Pathogen–Host Interaction Database (PHI) was used to predict potential pathogen-active proteins. A total of 4879 genes were predicted to play a role in pathogen–host interactions [51].

Cytochrome P450 (CYP450) is a large family of proteins that use heme iron as a coenzyme [52]. They catalyze the oxidation of a wide range of substrates and are involved in the metabolism of endogenous and exogenous substances. In *D. mahothocarpus* GZU-Y2, a total of 909 CYP450 genes were predicted.

The fungal secondary metabolites, including toxins, are believed to contribute to the pathogenicity of numerous plant pathogenic fungi and are referred to as potential virulence factors [53]. To analyze the virulence-related genes of *D. mahothocarpus* GZU-Y2, putative proteins were compared with the Database of Fungal Virulence Factors (DFVF), which identified a total of 3371 genes as fungus-independent factors.

### 3.7. Analysis of Protein Subcellular Localization

Protein subcellular localization analysis predicted a total of 1919 signal peptides. Additionally, 3467 transmembrane proteins, 1431 secreted proteins, and 164 effector proteins were predicted (Table 1). The specific information about 46 annotated effector proteins (hypothetical proteins not included) is listed in Table 5.

### 3.8. Comparative Analysis

As can be seen in Table 6, we comparatively analyzed six additional fungal genomes (*D. eres* CBS 160.32, *D. aspalathi* MS-SSC91, *D. citri* Q7, *D. citri* ZJUD14, *D. citri* NFHF-8-4 and *D. capsici* GY-Z16). Annotations of these genomes are publicly available and have been used to compare genome size, GC content, BUSCO Completeness, and number of CA-Zymes. The main reasons for selecting these species were their importance as plant pathogens and the availability of their annotations in public databases and published works [54]. Overall, the genomic characteristics of the analyzed species varied in terms of genome size, GC content, BUSCO Completeness, and number of CA-Zymes. The number of predicted genes ranged from 14,425 (*D. capsici* GY-Z16) to 16,499 (*D. eres* CBS 160.32), and BUSCO analysis verified assembly completeness and analyzed differences in the number of CA-Zymes. This analysis showed differences between disease genomes of different plants, such as *Camellia oleifera*, blueberry [55], soybean [56], citrus [57,58], and walnut [59], caused by *Diaporthe* species.

## 4. Discussion

**Figure 9:**
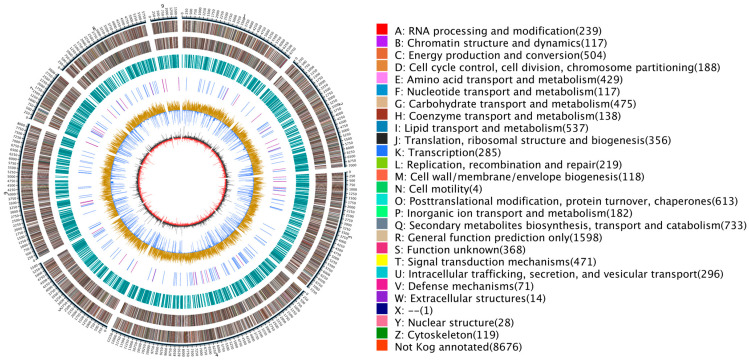
Genomic landscape of *D. mahothocarpus* GZU-Y2 visualized using Circos software (version 0.69-9). The outermost circle represents the location coordinates of the contig 1–9 of the genome sequence.

The distribution of plant diseases caused by *Diaporthe* is global, with a wide range of reported hosts, including Camelliaceae, Leguminosae, Walnutaceae, Rosaceae, Lacertaceae, and Vitaceae. *C. oleifera* is a very important woody edible oilseed tree species, and *D. mahothocarpus* GZU-Y2 is an important pathogen on *C. oleifera*, but its genome has not been previously characterized. In the previous study, we isolated and identified *D. mahothocarpus* GZU-Y2, the strain responsible for leaf blight disease in *C. oleifera*, from infected leaves. Here, in the present study, we sequenced, assembled, and predicted the *D. mahothocarpus* GZU-Y2 genome. Moreover, the high quality of the *D. mahothocarpus* genome sequence was demonstrated by the Contig N_50_ of 7,066,871 bp. Based on the results of genome assembly and annotation, the genome landscape is shown in Figure 9.

By comparing the genome of *D. mahothocarpus* GZU-Y2 with other genomic data, it was found that there were differences in gene structure, especially in genome size and number of coding sequences, which were significantly smaller in *D. mahothocarpus* GZU-Y2 than in *D. eres* CBS 160.32 and *D. citri* Q7. The reasons for this result may be the different number of genes and gene duplication events in *Diaporthe* species. And there were also differences in BUSCO Completeness as well as GC Content in different genomes; the results are shown in Table 6 [55,56,57,58,59,60]. Based on the Carbohydrate-Active enZYmes Database (CAZymes), we investigated CAZymes in the *D. mahothocarpus* GZU-Y2 genome in more detail.

It was found that the genome of *D. mahothocarpus* GZU-Y2 was found to contain 441 Glycoside Hydrolases (GHs), 134 Glycosyl Transferases (GTs), 36 Polysaccharide Lysylases (PLs), 205 Carbohydrate Esterases (CEs), 237 Auxiliary Active enzymes (AAs), and 102 Carbohydrate-binding related enzymes (CBMs), as predicted. This strain has a high capacity to disrupt plant cell walls during infection, as indicated by multiple GHs, GTs, and PLs. Plant cell hydrolases of phytopathogenic fungi are key causative factors in breaking down cell walls and establishing infection and nutrient growth [61]. Interestingly, the number of CAZymes number is greater than *D. eres* CBS 160.32 and *D. capsici* GY-Z16 and less than *D. citri* Q7 and *D. citri* ZJUD14, shown in Table 6. The amplification of relevant cell wall degrading enzymes in the genome of *D. mahothocarpus* GZU-Y2 is likely to be an important factor in the infection of *C. oleifera* [62]. The combination of multiple AAs and CBMs is also expected to have a significant impact on *D. mahothocarpus* GZU-Y2 [63,64].

Furthermore, by annotating the genome of *D. mahothocarpus* GZU-Y2, we predicted a total of 909 CYP450 genes for this strain. It was found that CYP450 genes can be involved in many important cellular pathways, including primary and secondary metabolism, toxin production, and detoxification, and can catalyze oxidation reactions of a variety of substrates and participate in the metabolism of endogenous and exogenous substances. The identification of these genes provides research and development of biocides specific to *D. mahothocarpus* GZU-Y2 certain support [65]. Additionally, 4879 PHI genes were predicted to be involved in pathogen-host interactions, while 3371 genes were identified as fungal-independent factors. These findings provide insights into the mechanisms of infection and aspects of the pathogenicity of many phytopathogenic fungi [66,67]. The protein subcellular localization analysis predicted a total of 1919 signal peptide genes, 3467 transmembrane proteins, 1431 secreted proteins, and 164 effector proteins. The study predicted 135 genes associated with the transporter protein TCDB and found that the expression and changes of these genes were linked to the development of the disease.

## 5. Conclusions

Generally, a genomic resource of *D. mahothocarpus* GZU-Y2 causing *C. oleifera* leaf blight was provided in the present study, which is also compared with other related genomes. We found that the genome of *D. mahothocarpus* GZU-Y2 has many plant cell wall degrading enzymes, a virulence and protein secretion system associated with infestation, and focused on annotating and analyzing genes related to pathogenesis. Several related virulence factors can manipulate the host response and induce plant cell death, thereby favoring colonization by the pathogen. The rich results of sequencing and analyzing the whole genome of *D. mahothocarpus* GZU-Y2 of *C. oleifera* leaf blight provide an opportunity to understand further the characteristics of *C. oleifera* leaf blight fungi and a new starting point and idea for researching new control measures. Therefore, future studies using Dual RNA sequencing technology to clarify both host and pathogen transcriptomes may provide better insight into the process of pathogen infection and host defense mechanisms.

## Figures and Tables

**Table 1:** Genomic characteristics of *D. mahothocarpus* GZU-Y2.

	Features	Values
Reads features (PacBio)	Total read number (G)	6.92040043
	SeqNum	809,585
	SumBase (G)	6.92040043
	N50Len	9545
	MeanLen	8548
	MaxLen	45,409
Genome features	Predicted genome size (Mbp)	58.97
	Complete BUSCOs (%)	97.93
	Complete and single-copy BUSCOs (%)	97.24
	Complete and duplicated BUSCOs (%)	0.69
	Fragmented BUSCOs (%)	0
	Missing BUSCOs (%)	2.07
	Total Lineage BUSCOs	290
	GC content (%)	50.65
	Contig Length (bp)	58,973,678
	Contig Number	62
	Contig N50 (bp)	7,066,871
	Contig N90 (bp)	5,516,847
	Gaps Number	0
	Repeat sequence (%)	3.22
	Protein-coding genes	15,918
	Number of non-coding RNA	577
	Pseudogene number	4
	Protein Sequence and Transporter Protein Classification Database (TCDB)	135
	Pathogen host interactive genes	4879
	Cytochrome p450 Engineering Database	909
	Fungal virulence factors	3371
	Carbohydrate-active enzymes	1058
	Signal peptide	1919
	Transmembrane protein	3467
	Secreted protein	1431
	Effector protein	164

**Table 2:** Statistics of repeated sequence prediction.

Type	Number	Length (bp)	Percentage (%)
ClassI	246	593,229	1.01
ClassI/LINE	20	1337	0.00
ClassI/LTR/Copia	70	106,154	0.18
ClassI/LTR/Gypsy	28	3848	0.01
ClassI/PLE|LARD	57	209,844	0.36
ClassI/TRIM	11	3783	0.01
ClassI/Unknown	60	268,324	0.45
ClassII	153	16,242	0.03
ClassII/Helitron	5	381	0.00
ClassII/MITE	45	9075	0.02
ClassII/TIR	85	5974	0.01
ClassII/Unknown	18	1183	0.00
PotentialHostGene	58	480,745	0.82
SSR	738	354,092	0.60
Unknown	1693	571,362	0.97
Total	1195	1,901,760	3.22

**Table 3:** Basic information statistics of the predicted genes.

Features	Values
Number of protein-coding genes	15,918
Total length of protein-coding genes	33,450,242
Average length of protein-coding genes	2101.41
Total exon length	29,284,507
Average length of exons	623.78
Number of exons	46,947
Average number of exons per gene	2.95
Total length of CDS	23,415,588
Average length of CDS	506.72
Number of CDS	46,210
Average number of CDSs per gene	2.9
Total length of intron	4,165,735
Average length of intron	134.25
Number of introns	31,029
Average number of introns per gene	1.95

**Table 4:** Characteristics of the representative gene clusters.

Scaffold ID	Gene Cluster	Start	End	Length (bp)	Type	Most Similar Known Cluster	Predicted Core Structure(s) *	Similarity (%)
ptg000001l	r1c1	141,995	181,332	39,338	NRPS	α-acorenol	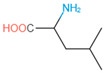	100
	r1c3	280,374	326,691	46,318	T1PKS	monascorubrin	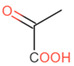	100
	r1c10	2,330,446	2,375,712	45,267	T1PKS	alternariol	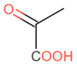	100
	r1c15	6,578,008	6,611,156	33,149	Terpene	koraiol	NA	100
ptg000006l	r6c12	6,311,961	6,332,174	20,214	Indole	sespendole	NA	83
ptg000007l	r7c3	2,657,144	2,703,633	46,490	T1PKS	wortmanamide A/ wortmanamide B	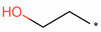	83
ptg000008l	r8c3	1,389,085	1,431,968	42,884	T1PKS	(-)-Mellein	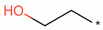	100

* Predicted core structure(s) is a rough prediction of core scaffold based on assumed PKS/NRPS colinearity; tailoring reactions are not taken into account.

**Table 5:** The information of the annotated effector proteins in the genome of *D. mahothocarpus* GZU-Y2.

Gene ID	Predicted Effector Proteins	Effector Probability
Dmahothocarpusptg000001lG002370.1	Probable endo-beta-1,4-glucanase D (Precursor)	0.691
Dmahothocarpusptg000001lG005880.1	Putative ec86 protein	0.739
Dmahothocarpusptg000001lG007160.1	Acetylxylan esterase 2 (Precursor)	0.787
Dmahothocarpusptg000002lG000540.1	CFEM domain-containing protein	0.713
Dmahothocarpusptg000002lG002080.1	Protein CAP22	0.558
Dmahothocarpusptg000002lG003240.1	Probable pectate lyase E (Precursor)	0.847
Dmahothocarpusptg000003lG000940.1	Putative bys1 domain protein	0.641
Dmahothocarpusptg000003lG003280.1	Lysine-specific metallo-endopeptidase	0.812
Dmahothocarpusptg000003lG006090.1	Putative sterigmatocystin biosynthesis peroxidase stcC	0.681
Dmahothocarpusptg000003lG006750.1	Cryparin (Precursor)	0.625
Dmahothocarpusptg000003lG008560.1	Putative sterigmatocystin biosynthesis peroxidase stcC	0.573
Dmahothocarpusptg000003lG008870.1	Phosphatidylglycerol/phosphatidylinositol transfer protein	0.787
Dmahothocarpusptg000003lG011640.1	CFEM domain	0.647
Dmahothocarpusptg000003lG016500.1	Putative transmembrane emp24 domain-containing protein 9 protein	0.650
Dmahothocarpusptg000003lG017280.1	Deoxyribonuclease NucA/NucB	0.710
Dmahothocarpusptg000003lG019450.1	Pectate lyase plyB (Precursor)	0.626
Dmahothocarpusptg000004lG000540.1	Putative carbohydrate-binding-like protein	0.558
Dmahothocarpusptg000004lG006290.1	short chain dehydrogenase	0.551
Dmahothocarpusptg000004lG006480.1	Pyranose dehydrogenase 3	0.697
Dmahothocarpusptg000004lG014360.1	Necrosis-inducing protein (NPP1)	0.650
Dmahothocarpusptg000004lG018200.1	Probable glutamine amidotransferase SNO1	0.552
Dmahothocarpusptg000004lG021380.1	Pathogen effector; putative necrosis-inducing factor	0.617
Dmahothocarpusptg000004lG022680.1	CoA binding domain	0.646
Dmahothocarpusptg000004lG024050.1	CVNH domain	0.886
Dmahothocarpusptg000004lG033200.1	Fungal fucose-specific lectin	0.618
Dmahothocarpusptg000005lG001070.1	Acetylxylan esterase-like protein	0.552
Dmahothocarpusptg000005lG005760.1	Cysteine-rich secretory protein family	0.737
Dmahothocarpusptg000005lG007250.1	Parallel beta-helix repeat protein	0.726
Dmahothocarpusptg000005lG008360.1	Cerato-ulmin (Precursor)	0.699
Dmahothocarpusptg000005lG012990.1	Putative exo-beta-glucanase protein	0.639
Dmahothocarpusptg000005lG014470.1	Pectate lyase F	0.838
Dmahothocarpusptg000006lG000360.1	IDI-2 precursor	0.814
Dmahothocarpusptg000006lG004760.1	Short chain dehydrogenase	0.862
Dmahothocarpusptg000006lG004870.1	Galactan endo-beta-1,3-galactanase (Precursor)	0.597
Dmahothocarpusptg000006lG006270.1	Aromatic peroxygenase	0.654
Dmahothocarpusptg000006lG008080.1	Phospholipase A2	0.570
Dmahothocarpusptg000006lG015010.1	Pathogen effector; putative necrosis-inducing factor	0.729
Dmahothocarpusptg000006lG022730.1	Chloroperoxidase-like protein	0.703
Dmahothocarpusptg000007lG001170.1	chitin deacetylase	0.586
Dmahothocarpusptg000007lG001450.1	Necrosis-inducing protein (NPP1)	0.652
Dmahothocarpusptg000007lG002990.1	Putative endo-beta-1,4-glucanase D	0.713
Dmahothocarpusptg000007lG003330.1	Pectate lyase D	0.68
Dmahothocarpusptg000007lG006760.1	Ribonuclease clavin (Precursor)	0.901
Dmahothocarpusptg000008lG001670.1	Hydrophobic surface binding protein A	0.840
Dmahothocarpusptg000008lG012160.1	Putative barwin-like endoglucanase protein	0.682
Dmahothocarpusptg000008lG014260.1	Putative chitin binding protein	0.657

**Table 6:** Comparison of genomic features among different *Diaporthe* species.

Species	Strain	Host	Next-Generation Sequencing	BUSCO Completeness (%)	Genome Size (Mb)	GC Content (%)	Predicted Genes	CAZymes	References
*D. mahothocarpus*	GZU-Y2	*Camellia oleifera*	Pacbio Sequel II	97.93	58.97	50.65	15,918	1058	This study
*D. eres*	CBS 160.32	Blueberry	Illumina HiSeq	98.40	60.80	47.60	16,499	859	[55]
*D*. *aspalathi*	MS-SSC91	Soybean	Illumina HiSeq 2000	97.60	55.00	51.00	14,962	ND	[56]
*D. citri*	Q7	Citrus	Illumina HiSeq	98.50	63.61	47.48	15,422	1624	[57]
*D. citri*	ZJUD14	Citrus	Illumina HiSeq	98.60	52.06	52.76	14,991	1581	[57]
*D. citri*	NFHF-8-4	Citrus	Illumina HiSeq	97.30	57.00	46.72	15,921	ND	[58]
*D. capsici*	GY-Z16	Walnut	PacBio Sequel	98.40	57.60	51.30	14,425	843	[59]

ND, no data.

## Data Availability

All data generated and analyzed in this study are included in this article. This Whole Genome Shotgun project has been deposited at GenBank under the accession JBBYIC000000000. The raw sequencing data and the assembly reported in this paper are associated with NCBI BioProject PRJNA1098494 and BioSample SAMN40910135 within the GenBank. The version described in this paper is version JBBYIC 010000000.
